# A China - Vietnam collaboration for public health care: a preliminary study

**DOI:** 10.1186/s41256-019-0116-0

**Published:** 2019-08-30

**Authors:** Si Zhu, Wenjun Zhu, Wenji Qian, Yao He, Jiayan Huang

**Affiliations:** 1 School of Public Health, Fudan University, Shanghai, China; 2 Key Laboratory of Health Technology Assessment, National Health Commission, Shanghai, China

**Keywords:** Health cooperation, Vietnam, China, Qualitative study

## Abstract

**Background:**

Vietnam has achieved significant results in terms of improving population health and sustainable development goals (SDGs). However, several public health problems remain such as high mortality deriving from non-communicable diseases (NCDs). As part of their long-standing relationship, Vietnam and China have collaborated in various health fields. The objective of this study was to examine the current situation of public health cooperation between Vietnam and China and suggest ways to deepen future co-operations.

**Methods:**

Between March and May 2018, we conducted 14 in-depth interviews with key informants from Vietnam and China. The inclusion criteria in this study were as follows: 1) researchers who worked at research institutes or universities and were engaged in health cooperation research, 2) officers who were from government agencies or international organizations (IOs) and had been involved in, or were familiar with the health cooperation projects between China and Vietnam. The interviews were audiotaped and transcribed verbatim, and then analyzed to identify current cooperation strategies and cooperation fields, as well as to provide suggestions for future collaborations.

**Results:**

Current health cooperation mechanisms between China and Vietnam include bilateral and multilateral cooperation such as ASEAN Plus Three (China, Japan and ROK), ASEAN Plus One (China), the Greater Mekong sub-region, and the Lancang-Mekong Cooperation. This health cooperation can be summarized in terms of health security, health development, and health-related innovation. The health cooperation priorities outlined by our informants consisted of unimplemented SDGs such as NCD problems and the public health system. A proposal for future health collaboration was to establish a triangular cooperation between China, IOs/non-government organizations (NGOs) and Vietnam.

**Conclusions:**

The existing cooperation between China and Vietnam in bilateral and multilateral levels has provided a good foundation for a deeper and more extensive future partnership. Key areas of future cooperation would be to achieve SDGs and solve NCD related problems, which can be accelerated through favorable cooperation and reliable partnerships. A triangular cooperation between China, Vietnam, and IOs/NGOs was considered as a suitable future mechanism.

## Background

Vietnam has made impressive progress in improving population health. In terms of life expectancy (LE), the average LE at birth was 76.3 years in 2016, which was similar to that of the Western Pacific Region (76.9) and much higher than the global average (72.0) [1]. In addition, Vietnam is well on the way to achieving its Millennium Development Goals (MDGs), highlighting its rapid progress in universal health. The maternal mortality ratio (MMR) was 54 per 100,000 live births in 2015 (i.e. a quarter of the global average, which is 216) which met the SDG Target 3.1 by 2030, to reduce the global maternal mortality ratio to less than 70 per 100,000 live births [1]. A similar pattern emerges in terms of under-five mortality (U5MR). In 2015 the U5MR was 21.6 per 1000 live births, which was about half of the global average (40.8), already meeting the SDG target [1]. With a remarkable decline in MMR and a dramatic halving of U5MR in recent decades, Vietnam’s maternal child care compares favorably with other countries with a similar per-capita income [2].

Despite the improved health-related SDG index scores, progress in Vietnam varies across individual indicators [3]. Five red health-related SDG indicators still remain,, all of which are in the public health domain [4]. SDG target 3.4 - the age-standardized death rate due to cardiovascular disease, cancer, diabetes, and chronic respiratory disease in populations aged 30–70 (per 100,000 population) - has declined steadily in Vietnam in the past three decades, with 0.55% per annum in the 1990s to 1.23% per annum in 2010s. The death rate from NCDs in Vietnam was still 427.7 (per 100,000 population) in 2016 [5]. The high NCD mortality has become an urgent problem in Vietnam, while in contrast globally, the reduction rate of NCDs has already met the SDG goal with the equivalent of a 32.5% reduction in NCD mortality [6].

Vietnam and China share a border and also have similar political regimes, cultures, and development policies, along with a long-standing friendship [7–9]. As bilateral friendship and cooperation are of great importance to both nations [10], a Vietnam-China cooperative partnership will likely deepen the win-win cooperation between the two countries, and also bring greater benefits for both countries and citizens. The development path of Vietnam and China’s health sector is also similar, thus the success or failure of the health sector is highly significant for both sides [11].

The two countries have collaborated in the health sector via both multilateral and bilateral mechanisms. Vietnam’s Ministry of Planning and Investment and China’s Ministry of Commerce signed a Memorandum of Understanding in 2017 regarding cooperation for public health care assistance in Vietnam [12]. Regional multilateral cooperation mechanisms include amongst others the Association of Southeast Asian Nations (ASEAN), ASEAN Plus Three (China, Japan and ROK) and ASEAN Plus One (China), the Greater Mekong sub-region (GMS), and the Lancang-Mekong River cooperation (LMC) [13]. The health cooperation began in the fields of health security (prevention and control of infectious diseases and health emergencies), health development (advanced health systems, maternal and child health, health assistance, and traditional medicine), and medical innovations (joint research projects and health industry) [13].

Recognizing that science and disease have no national borders, increasing attention has focused on global public health. At both the domestic and global levels, the emphasis of health cooperation has made a transition from MDGs to SDGs. Existing cooperative practice has shown that cooperation can effectively solve public health problems and enhance public health capabilities [14, 15]. In terms of cooperative opportunities, the Chinese government plans to launch a public health network in addition to policy research, hospital alliance, and health industry networks—under the broader goal of advancing the UN 2030 SDGs [16].

Health cooperation is an opportunity for both sides to strengthen the national public health capacity to address common and particular public health problems, including SDG targets. The aim of this research is thus to identify the current situation of Vietnam-China public health cooperation and to propose future collaborations. Our hope is that the results of this study will provide a reference for better and deeper cooperation in the future between Vietnam and China, and also between other counties with similar characteristics.

## Methods

### Study setting and participants

We collected qualitative data through in-depth interviews with key informants. Convenience sampling was used to select the participants. The inclusion criteria in this study were as follows: 1) researchers who worked at research institutes or universities and were engaged in health cooperation research, 2) officers who were from government agencies or IOs and had been involved in, or were familiar with the health cooperation projects between China and Vietnam. Qualitative interviews were conducted between March and May 2018 by trained interviewers through phone calls or Skype at Fudan University, Shanghai, China with 14 key informants from Vietnam and China. An email was sent to each informant inviting them to participate. Detailed information on the participants is presented in Table 1.

**Table 1:** Characteristics of interviews

Country	Organization	Interviews(n)
China	National health commission	3
China	Fudan University	1
China	Peking University	1
China	Chinese Academy of Social Sciences	1
China	WHO local office	1
Vietnam	Ministry of Health	2
Vietnam	Hanoi University of Public Health	2
Vietnam	WHO Vietnam office	1
Vietnam	UNFPA Vietnam office	1
Vietnam	UNICEF Vietnam office	1

### Data collection

Verbal informed consent was obtained from all participants before initiating the interviews. Each interview lasted 60 min and was moderated by the authors following a semi-structured, open-ended format. The questions focused on: (1) the current cooperation strategy; (2) the current fields of cooperation; (3) suggestions for future collaborations.

### Data analysis

The interviews were audiotaped and transcribed verbatim suing MS Word, after which they were reviewed and edited by the co-authors. NVivo 10 was used to organize and analyze the data.

### Ethics

The ethics committee of the School of Public Health of Fudan University approved the research. Interviewers were not permitted to ask participants for personal information. Information on the study was distributed to all participants. All participants clearly understood the purpose of the study and principles of voluntary participation.

## Results

### Current health cooperation

#### Bilateral cooperation

Cross-border collaborations between Vietnam and China have been operating for decades and are currently mainly negotiated by the two countries’ National Health Commissions. Non-governmental cooperation has continuously expanded, while bilateral government-to-government cooperative opportunities are limited, as expressed by one of our informants: “*Cooperation with Vietnam in recent years has been more under multilateral mechanisms and frameworks, including talks at international conferences.*” The ever-changing foreign-policy interests were mentioned by the key informants as one of the important influences for further cooperation.

Although health interventions are increasingly used to support foreign-policy objectives, foreign-policy interests may actually have impeded the development of health cooperation. Several key informants believed that funding sources may affect the desire for cooperation. However, scientific cooperation research programs between Vietnam and China researchers have been consistently developed. For example, in the past few years, two of the interviewees have joined a joint research project on maternal and child health. As one interviewee stated: “*The similarity of the health system in terms of the development path provides a necessary basis for scientific research cooperation*.” Note: hereafter all sentences in italics are quotations from our interviewees.

#### Multilateral cooperation

Being one of the most important organizations in Southeast Asia, the ASEAN framework has provided a good platform for strategic consultation: “*The cooperation mechanism provided by ASEAN member countries is mature and perfect which benefits further cooperation*.” Two levels of cooperations were mentioned under ASEAN Plus Three (China, Japan and ROK) and ASEAN Plus One (China). One is the ASEAN Health Ministers’ Meeting (AHMM), which is held every two years and which provides a platform for political commitment, highlighting the willingness to continue to strengthen and promote cooperation. The other is the Senior Officials’ Meeting on Health Development (SOMHD) held each year, through which information is shared: “*AHMM is more of a political commitment, showing that China and ASEAN countries are very willing to continue deepening and promoting cooperation in the health field. SOMHD plays the role of information sharing, including the sharing the work of each country at an official level, i.e. what they have done for the ASEAN countries, which reflects their influence and ability.*” In addition, there are also several technological cooperation mechanisms, for example the ASEAN Expert Group on Communicable Diseases (AEGCD) was formed to work together for the prevention and control of communicable diseases. However, a few participants pointed out that the limited funding provided by the ASEAN may lead to some problems: “*A direct reporting network system of communicable diseases has been established, but operations have faced huge problems due to a lack of financing*”. Although funding is limited, the existing cooperation mechanism has made it easier to carry out work smoothly and to achieve the ultimate goal: the establishment of mutual trust.

Another important multilateral cooperation mechanism is the Greater Mekong sub-region (GMS), established in 1992 with the assistance from the Asian Development Bank (ADB). Its member countries including Vietnam, China (specifically Yunnan Province and Guangxi Zhuang Autonomous Region), Cambodia, Lao People’s Democratic Republic, Myanmar, and Thailand. In addition, the Mekong Basin Disease Surveillance (MBDS) network was established through a memorandum of understanding signed by the Ministers of Health of these six countries. Interviewees reported that the lack of supervision and funding could make it difficult for the network to ensure normal functions: “*The current problem is that we do not have the right leaders. Moreover, the financing ability of MBDS is very poor, which results in huge operational problems*. *It has raised only 20,000-30,000 US dollars per year, which is only enough to guarantee the existence of the secretariat. Right now the only thing MBDS does is to send regular newsletters to the member countries.*” Despite the challenges, interviewees believed that the network could be reactivated by sustainable capital support and effective campaigns. They also perceived that the activated network had a positive impact not only on the cross-border prevention and control of emerging and re-emerging infectious diseases but also on the relations between the member countries: “*I think the key factor to solve the problem is ‘money’. Moreover, it requires long-term strategic financial support. In order to reactivate the network and attract members’ attention, several activities are needed. For example, when there is a new infectious disease outbreak, we can share information through MBDS, then information can be transferred to all member countries. Members will then see MBDS as being very useful and share freely information about it.”*

The interviewees pointed out that the Lancang-Mekong Cooperation (LMC), a new multilateral cooperation mechanism initiated by China in 2015, showed great potential for development. The member countries include China, Vietnam, Cambodia, Thailand, Laos, and Myanmar. The LMC framework is primarily focused on the management of water resources, but as it is based on mutual trust and benefit, its scope has been extended to people’s livelihood including environmental health, health promotion, and public health: “*The control of water resources is closely related to the control of environmental health and infectious diseases, which can win the hearts and minds of local residents. Thus the prevention and control of waterborne infectious diseases are beneficial to the management of water resources*.” One of the priorities is the prevention and control of emerging and re-emerging infectious diseases, especially the establishment and improvement of early warnings and joint surveillance mechanisms.

One of the participants highlighted the similarity of and the relationships among these cooperative frameworks. Although involving the same countries and sharing similar cooperation fields, these frameworks are neither exclusive nor in competition, and they differ in terms of the underlying multilateral negotiations.

### Cooperation field

The key informants all agreed that current cooperations essentially focus on three fields: health security, health development, and health-related innovation. With the development and constant changes in health needs, the priority areas for regional cooperation are also continually changing: “*From health emergencies to health systems, and then from conventional chronic diseases, to the overall needs of the population along with healthy aging.*”

### Health security (prevention and control of infectious diseases and health emergencies)

Several key informants highlighted that there are cooperation projects regarding infectious diseases which are carried out on the borders between the two counties. Current projects involve AIDS, tuberculosis, and cholera. With regard to China, the CDC in the nearby provinces is responsible for the cooperation projects. Activities include seminars on infectious disease monitoring and meetings on the exchange of prevention and control technologies: *“At present, China has a cooperation project with Vietnam on the prevention and control of border infectious diseases, which is under the responsibility of Guangxi CDC and Yunnan CDC (border provinces). The project mainly includes personnel training and natural disaster assistance.”*

### Health development (strengthening health systems, maternal and child health, health assistance and traditional medicine)

Some key informants mentioned that the two countries have long-established maternal and child health cooperative projects as both countries follow a family planning policy: “*The Office for Population and Family Planning of Vietnam, which is responsible for Vietnam’s family planning policy, is in close contact with us (China)*. *After the health reform in Vietnam in 2013, the health and family planning authorities in Vietnam and China have exchanged visits every year*. *In recent years, due to financial constraints, the contacts have been less frequent.*” In addition, participants reported Vietnam’s interests in the Chinese experience of health care reform: “*Every year, Vietnam’s government sends one or two delegations to China to study health care reform policies, covering a wide range of fields, including rural health, maternal and child health and human resources.*” They also mentioned that traditional Chinese medicine has won high recognition from ASEAN countries and cooperation projects on the subject have already been launched.

### Health related innovation (joint research and health industry)

Some key informants considered health-related innovations, including joint research projects and health industry, as future development priorities. Several companies have set up a number of projects across Southeast Asia countries. “*The Beijing Genomics Institute has started health-related innovation projects mainly related to public welfare, and its genetic sequencing products are used for the early screening of thalassemia and deafness in Southeast Asia.*”

### Suggestions for future collaboration

#### Cooperation priorities

The interviewees felt that promoting health development strategies should be the first step in any collaboration process, followed by a willingness to set priorities in order to decide the exact fields of collaboration. In fact the informants emphasized that it would be significantly easier to collaborate if both sides share the same interests and needs.

#### China’s priorities

SDGs were mentioned as the collaboration priorities from the Chinese perspective: “*The ultimate goal of China’s contribution is to achieve global SDG. We maintain bilateral cooperation in order to support the achievement of global goals*.” Interviewees indicated that China’s Belt and Road (also called the Silk Road) Initiative was an effective way to achieve SDGs globally: *“Under the Belt and Road Initiative, the “Belt and Road High Level Meeting for Health Cooperation: towards a Health Silk Road” was launched and the implementation plan was designed to achieve the goal of SDG and contribute to global development.”* Also, NCDs and aging problems were identified as priority cooperation objectives. *“Infectious diseases are staged problems, and chronic diseases are long-term problems. In addition, issues regarding aging need to be added.”*

#### Vietnam’s priorities

The SDG strategy in Vietnam was mentioned by our informants as the national partnership cooperation framework, specifically including universal health coverage and health system establishment. In terms of health priorities in Vietnam, most respondents reported that the problems of NCDs including cardiovascular disease, cancer, diabetes, and chronic respiratory diseases were urgent and that collaboration with China was important, especially for management at the community level.

The Vietnamese still have various health problems related to smoking and drinking, especially men: *“Vietnamese men like drinking beer and alcohol. Vietnam is one of the countries with the highest level of alcohol consumption. And also there is a problem with smoking. Smoking is still very high among men.”* Current NCD related problems mentioned by interviewees included community-level health care services and two-way referrals between hospitals and community healthcare systems. *“The problem of NCDs is becoming more urgent. In particular, the population is aging in Vietnam as fast as it is in China. So the government really needs to focus on strategies. People with hypertension, metabolism diseases and conditions, diabetes, mental health, need to come to the clinic or health facility many times each month to get their medicine, and for check ups. So the government set up the system to manage the whole thing, they want to transfer the task from the hospital to the community. I think the situation is very similar in China.”*

In addition to the priority cooperation areas from Vietnam’s perspective, the most frequently mentioned topic during the interviews was the public health system. It was also noted that Vietnam has focused on establishing the CDC for years and is interested in China’s CDC model: *“I think there is a huge need [for this to happen](by merging some departments to become the CDC). This issue was already discussed 20 years ago, but it takes some time for the government to change.”* For instance, at the end of 2017, a national Vietnamese team visited the Chinese CDC to learn about the construction of China’s disease control system. *“They learnt about your model in terms of the CDC, in terms of the management. But maybe now they don’t go up to the ministry level, they go directly to the institutions. They learn the CDC model.”*

#### Forms of cooperation

A common perception was that health personnel training was effective: *“I advocate capacity training in the early stages of cooperation, which can cover different fields in various forms on a voluntary basis.”* Agency visiting, experience sharing and course discussions were all the suggested forms of long-term or short-term health personnel projects. Participants also suggested that medical consultation services provided inspiration for involvement and that their value in terms of long-term cooperation was immense. *“I also advise expert consultation to solve specific problems. If the partners agree with the solutions and methods proposed by the experts, we can go ahead with subsequent projects.”* There were concerns that without the guarantee of funds and close cooperation, pilot projects may not fully succeed.

#### Cooperation strategies

The triangular cooperation among China, IOs/NGOs and Vietnam was recommended as an effective approach to future collaboration: *“China - partner countries - IOs is now basically recognized at the official level.” “The NGOs represented by the Bill & Melinda Gates Foundation have developed very fast in the past decade, and their investment in health cooperation is even more than that in many countries.”* It seems that triangular cooperation was not yet well developed but has demonstrated its potential. Many interviewees saw triangular cooperation as a new trend deserving further exploration. One participant mentioned that the Bill & Melinda Gates Foundation cooperates with the International Health Exchange and Cooperation Center NHC PRC to support international health cooperation related research.

## Discussion

### Further consolidation and development of bilateral cooperative partnerships

We believe that this is the first qualitative study to explore public health collaboration between Vietnam and China. Vietnam and China have established cooperation mechanisms at bilateral and multilateral levels for years and several successful cooperative projects in various fields have been carried out. Vietnam has witnessed a rapid health development over the last few decades. The most important focus of China’s long-term security is health [17]. Health is also one of the important components in China’s Belt and Road Initiative, which was proposed by the Chinese government and has the participation of various countries, aimed at an extensive commitment to global health development [16, 18, 19]. The initiative provides a rare opportunity to further consolidate cooperative partnerships with the gradual establishment of mutual trust and reciprocal cooperation.

Our findings also show that stable political relations are the basis of bilateral cooperation, while fostering trust and increasing investment promotes the development of bilateral cooperation. On the other hand, there is a clear need for monitoring and evaluation mechanisms. Further research is needed to measure the effectiveness and outcomes of such collaboration mechanisms, therefore maintaining the sustainability and high efficiency.

### Deepen the willingness for cooperation and identify the areas of cooperation

Health security cooperation such as health protection against infectious diseases is essential for bordering countries to achieve economic goals [16, 17], and also benefits both countries and especially border residents. Cooperation in the field of both health development and health-related innovation has made clear progress thereby establishing a solid foundation for future cooperation. Both sides should continue their dialog and interaction in existing areas, and at the same time, explore new and potential areas for the future. As the areas of cooperation have expanded and forms of cooperation have increased, Vietnam and China can further can find new ways to strengthen their mutual cooperation in order to improve national and global health.

In order to stimulate long-term cooperation and confidence-building, key areas of future cooperation need to be identified. Our study results show that the main aspects of cooperation should comply with national future development plans and current priorities. With economic and social development, both people’s health needs and the country’s health development priorities are constantly and rapidly changing [20]. Plans for the health development of both countries have targets linked to SDGs which represent global policy goals. In our study, SDGs were found to be the priority of China and Vietnam’s health development. Progress for future SDG achievements can be accelerated in the coming years through health cooperation.

Vietnam has been hit by health threats from both infectious and NCDs like many low-middle income countries due to the spread of risk factors associated with globalization and urbanization [21, 22]. Both countries, therefore, are in a crucial position and share a strong imperative to solve the problem of NCDs.

Global health friendships have focused on product-oriented interventions and communicable diseases for several decades. However, NCDs and life-style risk factors could now become the core activities [23]. China has not only made good progress and accumulated experience in developing and implementing NCD prevention strategies at the community level [24], but has also faced challenges that resonate with the difficulties experienced in many developing countries including Vietnam. Health data and programs that incorporate sex-specific dimensions have become of increasing concerned [6], especially in Vietnam whose male population generally experiences a greater toll than the females in terms of adult mortality rates [25]. In addition, various forms of increasing cooperation will likely stimulate new dialogs regarding policy and research in order to provide a basis for future cooperation between Vietnamese and Chinese policy makers which can be translated into timely policy decisions.

### Strengthen the top-level design of cooperation

Our study also highlights the need for multilateral cooperation, especially triangular cooperation among China-Vietnam-IOs/NGOs. Major IOs in Vietnam include WHO, UNICEF and several other UNDG Agencies which have participated in the One UN Initiative since 2006 [26]. Through triangular cooperation, the stakeholders involved can benefit mutually as a result of the experiences and learning exchanged, thus increasing the sustainability of the cooperation results [27, 28]. Especially in terms of public health problems, NGOs have played a larger role than expected [29]. As Vietnam is about to entering middle-income countries, IOs started to divest. At the same time, direct foreign investment in SDGs declined sharply [30, 31]. China is likely to assume a greater contributory role in multilateral programs and pursue increasing cooperation with multilateral initiatives [16]. Both sides should also continue to unpack the conceptual gaps and facilitate the building of a long-term stable triangular collaborative relationship.

### Limitations

Research on the cooperation between China and Vietnam is very limited, especially in the field of public health. Bias cannot easily be avoided due to the convenience sampling. However, the participants in this study were extremely keen to participate and may thus have had particularly strong views regarding the public health collaboration between the two countries. Qualitative research is hard to replicate, and thematic analysis may limit the generalizability of the data and the external validity of the results [32]. Nevertheless, these results provide useful information to help guide the implementation of China - Vietnam collaboration for public health care in the future.

## Data Availability

The datasets generated and/or analyzed during the current study are not publicly available because the study uses primary data of interviews with participants.
